# Arterial spin labeling signal in the CSF: Implications for partial volume correction and blood–CSF barrier characterization

**DOI:** 10.1002/nbm.4852

**Published:** 2022-11-09

**Authors:** Léonie Petitclerc, Lydiane Hirschler, Balázs Örzsik, Iris Asllani, Matthias J. P. van Osch

**Affiliations:** ^1^ C.J. Gorter MRI Center, Department of Radiology Leiden University Medical Center Leiden The Netherlands; ^2^ Leiden Institute for Brain and Cognition (LIBC) Leiden The Netherlands; ^3^ Department of Radiology Leiden University Medical Center Leiden The Netherlands; ^4^ Clinical Imaging Science Center, Department of Neuroscience University of Sussex Brighton UK; ^5^ Department of Biomedical Engineering Rochester Institute of Technology Rochester NY USA

**Keywords:** arterial spin labeling, blood–CSF barrier, brain clearance, glymphatics, neurofluids, partial volume correction

## Abstract

For better quantification of perfusion with arterial spin labeling (ASL), partial volume correction (PVC) is used to disentangle the signals from gray matter (GM) and white matter within any voxel. Based on physiological considerations, PVC algorithms typically assume zero signal in the cerebrospinal fluid (CSF). Recent measurements, however, have shown that CSF‐ASL signal can exceed 10% of GM signal, even when using recommended ASL labeling parameters. CSF signal is expected to particularly affect PVC results in the choroid plexus. This study aims to measure the impact of CSF signal on PVC perfusion measurements, and to investigate the potential use of PVC to retrieve pure CSF‐ASL signal for blood–CSF barrier characterization. In vivo imaging included six pCASL sequences with variable label duration and post‐labeling delay (PLD), and an eight‐echo 3D‐GRASE readout. A dataset was simulated to estimate the effect of CSF‐PVC with known ground‐truth parameters. Differences between the results of CSF‐PVC and non‐CSF‐PVC were estimated for regions of interest (ROIs) based on GM probability, and a separate ROI isolating the choroid plexus. In vivo, the suitability of PVC‐CSF signal as an estimate of pure CSF was investigated by comparing its time course with the long‐TE CSF signal. Results from both simulation and in vivo data indicated that including the CSF signal in PVC improves quantification of GM CBF by approximately 10%. In simulated data, this improvement was greater for multi‐PLD (model fitting) quantification than for single PLD (~1–5% difference). In the choroid plexus, the difference between CSF‐PVC and non‐CSF‐PVC was much larger, averaging around 30%. Long‐TE (pure) CSF signal could not be estimated from PVC CSF signal as it followed a different time course, indicating the presence of residual macrovascular signal in the PVC. The inclusion of CSF adds value to PVC for more accurate measurements of GM perfusion, and especially for quantification of perfusion in the choroid plexus and study of the glymphatic system.

## Graphical abstract

ASL signal in the CSF is non‐zero, and including this in partial volume correction leads to better estimates of GM CBF. Differences in GM signal between CSF‐PVC and non‐CSF‐PVC averaged 10% over all the GM, and 30–40% in the choroid plexus. CSF‐PVC is especially recommended for choroid plexus perfusion measurements.

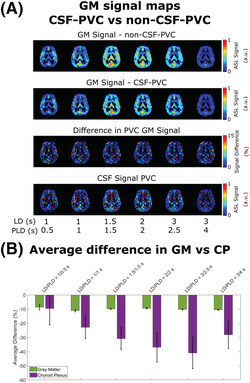

**Abbreviations**
aBV: arterial blood volume
ASL: arterial spin labeling
ATT: arterial transit time
BCSFB: blood–CSF barrier
CSF: cerebrospinal fluid
CSF‐PVC: PVC with CSF signal ≠ 0
GM: gray matter
GM_prob_
: probability, or partial volume, of GM in a given voxel
LD: label duration
non‐CSF‐PVC: PVC with CSF signal = 0
PLD: post‐labeling delay
PV: partial volume
PVC: partial volume correction
ROI: region of interest
SNR: signal‐to‐noise ratio
T_1_w: *T*_1_ weighted
*T*_bl→CSF_
: exchange time from blood to CSF
TPM: tissue probability map
WM: white matter

## 1 INTRODUCTION

Arterial spin labeling (ASL) is a non‐invasive perfusion method, which typically uses coarse image resolution to compensate for its inherently low signal‐to‐noise ratio (SNR). Consequently, any given voxel in an ASL image contains signal from a number of different tissue contributions, introducing bias to the resulting perfusion measurements in any ASL quantification method. For this reason, partial volume correction (PVC) algorithms have been proposed as a means to extract the pure perfusion parameters for gray and white matter tissue, separately.^1^, ^2^, ^3^ In its original form, PVC uses voxel‐wise volume fraction estimates for each tissue type as extracted from high resolution anatomical imaging, and, assuming that perfusion is the same for neighboring voxels, solves a system of linear equations to calculate pure gray matter (GM) and white matter (WM) perfusion, defined as the perfusion value that would be measured if the voxel contained 100% GM or WM. Traditionally, the cerebrospinal fluid (CSF) contribution to the total ASL perfusion signal is assumed to be zero, and only GM and WM perfusion values are extracted. However, we have shown in a recent study that labeled water does in fact exchange into the CSF to create a measurable ASL signal, and that this signal is present throughout the brain, outside of the widely accepted exchange sites in the choroid plexuses.^4^ With such a broad spatial distribution and an intensity of about 10% of the GM signal at clinical perfusion ASL parameters, CSF‐ASL signal may significantly impact PVC results throughout the brain. In particular, blood perfusion in the choroid plexus itself has become the topic of a number of recent studies,^5^, ^6^, ^7^, ^8^ owing to its important role in the brain waste clearance system. There are a few factors that make this structure uniquely relevant to this study: first, it is a small structure bathing in CSF, meaning that partial voluming effects with CSF are bound to be large, and second, it is a known site of CSF production, and therefore ASL signal in the CSF immediately surrounding it is undoubtedly non‐zero. Consequently, additional attention was devoted in the current study to the effect of CSF on PVC in the choroid plexus.

Moreover, the inclusion of CSF contributions in the PVC model may offer the opportunity to isolate the CSF signal and thus quantify water transport across the blood–CSF barrier (BCSFB) without modifications to existing ASL sequences. This would be especially useful as current CSF‐ASL measurements require ultra‐long echo times (TE) to isolate the long‐*T*_2_ CSF signal, making this sequence time consuming to acquire and infeasible in a clinical setting. Employing a PVC algorithm to retrieve CSF signal without the use of long echo times not only may allow for rapid assessment of the BCSFB in future studies, but also this technique could be applied retrospectively to already acquired data. Such considerations formed the impetus for this study, which has two primary aims:

1. to assess the effect of CSF‐ASL signal on apparent GM cerebral blood flow estimates using PVC algorithms that previously neglected the contribution of CSF;
2. to investigate the use of PVC to isolate the CSF‐ASL signal as an alternative to ultra‐long TE acquisitions.

## 2 METHODS

### 2.1 Simulation of CSF‐ASL data

To test the impact of CSF‐ASL signal on pure GM perfusion measurements, a simulated dataset was created as follows: GM and WM CBF were set to 60 and 20 mL/100 g/min respectively.^9^ Arterial transit times (ATTs) were set to 1.0 and 1.2 s for GM and WM, respectively.^4^, ^9^ The *T*_1_ of blood was set to 1,650 ms,^10^ and the labeling efficiency, *α*, to 0.85. For the purposes of this simulation, the blood–brain partition coefficient, *λ*, was assumed to be 1, and the equilibrium magnetization of tissue, *M*_0_, was set to 1 (as it simply acts as a scaling factor). Pure WM ASL signal was computed for all label durations (LDs) and post‐labeling delays (PLDs) of the MRI experiment using the Buxton model,^11^ and at all TE values with the *T*_2_ of WM set to 60 ms.^12^ CSF and GM signals and especially the transport of label into the CSF compartment were simulated using the two‐compartment exchange model presented in, Reference 4 which includes a CSF (*T*_1_ and *T*_2_ of 4300 and 1500 ms, respectively)^13^, ^14^ and a blood + GM compartment (without macrovascular blood, average *T*_2_ of 100 ms).^12^, ^15^ Simulated data were created for 10 different values of the exchange parameter *T*_bl→CSF_ (40–85 s, 5 s step) centered around the average of 60 s as previously found in healthy volunteers.^4^ This allows for the estimation of the impact of CSF‐ASL signal for a range of conditions from little/slow exchange (long *T*_bl→CSF_) to high/rapid exchange (short *T*_bl→CSF_). To create simulated ASL signal maps, the pure GM, WM, and CSF signals are multiplied by standard tissue probability maps (TPMs) from SPM12 (Wellcome Trust Centre for Neuroimaging, UCL, London, UK) for the corresponding tissue/fluid type. These maps were then downsampled to a typical ASL resolution, from 1.5 mm isotropic to 3 × 3 × 6 mm^3^, to create partial volume (PV) maps. Finally, white Gaussian noise with an SNR of 20 (based on the average signal for all echo times and LD/PLDs) was added. All simulations and data analysis were performed in MATLAB R2019b (MathWorks, Natick, MA, USA).

### 2.2 MRI acquisition

Thirteen healthy volunteers (11 female, ages 21–67 years, median 28 years) were scanned after providing written informed consent following institutional review board regulations in accordance with the Declaration of Helsinki. All scans were performed on a Philips Achieva XT 3 T system (Philips, Best, The Netherlands). The multi‐echo pCASL sequence is the same as the one we employed in our previous publication,^4^ and we refer the reader to this for more details. Briefly, six separate sequences were acquired with LD/PLD = 1/0.5, 1/1, 1.5/1.5, 2/2, 3/2.5, 3/4 s, each using a multi‐echo gradient‐and‐spin‐echo (GRASE) readout with eight TE values of 10 + 261*n* ms (*n* = 0–7). For quantification, an *M*_0_ image (same readout as the ASL sequences, but without labeling) was acquired with TR = 10 s and TE = 10 ms to ensure maximum recovery of the longitudinal magnetization of tissue. A high resolution *T*_1_‐weighted (T_1_w) MP‐RAGE image was acquired (TR/TE = 9.8/4.6 ms, 0.875 × 0.875 × 1.2 mm^3^ resolution anterior/posterior × right/left × foot/head, scan time 5 min) for the estimation of tissue PVs. The total length of the scan session was approximately 1 h.

### 2.3 Data analysis

**FIGURE 1:**
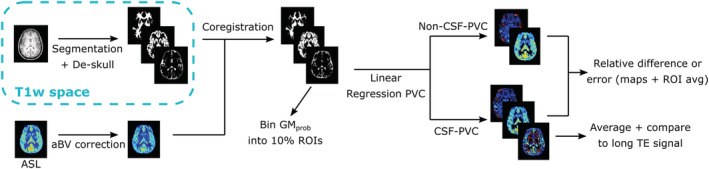
Schematic representation of PVC algorithm pipeline for in vivo signal. For simulated data, no aBV correction is performed, and error is calculated with respect to the ground truth.

The image analysis pipeline described below is shown schematically in Figure 1.

#### 2.3.1 Image pre‐processing

ASL control and label images were pair‐wise subtracted to extract the ASL signal at all LD/PLDs and TE values. The T_1_w image (high resolution) was segmented using SPM (six tissue types) to compute the probabilities for GM, WM, and CSF in every voxel, which were employed as estimates for the PV. Three tissue masks were created from these maps with a tissue probability threshold of more than 30%; these masks were combined into one intracranial mask (i.e., if one or more of the three tissue probabilities for a voxel exceeded 30%, it was classified as part of the intracranial volume), to remove any background signal and the skull from the T_1_w image. This “de‐skulled” image was coregistered to the *M*_0_ scan to transform it to the ASL space (all ASL scans and *M*_0_ have the same geometry and planning, and we assume no movement between them). Subsequently, the same transformation matrix was applied to the TPMs for GM, WM, and CSF, resulting in PV estimates for each tissue in every voxel in ASL space as input for the PVC algorithm.

#### 2.3.2 ASL processing and removal of macrovascular signal

The in vivo ASL images were smoothed with a Gaussian kernel of 3 × 3 voxels and *σ* = 2 voxels before any data processing. Since the original PVC‐algorithm does not include a macrovascular component, and neither does the simulated dataset, we opted to exclude macrovascular signal from in vivo data as much as possible by fitting the function

(1) $$∆M=∆M_{\text{Buxton}}+∆M_{\text{macro}}$$

with

$$∆M_{\text{macro}}=\left\{\begin{array}{c} 2\alpha M_{0}e^{-\mathrm{ATT}/T1b}\mathrm{aBV},\text{if}\mathrm{ATT}\leq t<\mathrm{LD}+\mathrm{ATT}\\ 0,\text{otherwise} \end{array}\right.$$

to the signal in the first echo using the MATLAB function lsqcurvefit, with ∆*M*_Buxton_ from the Buxton perfusion model.^11^ The arterial blood volume (aBV) was voxel‐wise estimated using Equation 1 and then removed from the original ASL signal. All *M*_0_ values used for this and subsequent analyses were an average over the GM (single value for each subject) divided by the blood–brain partition coefficient *λ* = 0.98 mL/g.^16^ T_1b_ represents the T_1_ relaxation time of arterial blood (set to 1650 ms).

#### 2.3.3 Linear regression PVC

The linear regression PVC algorithm has been described in detail elsewhere.^1^ In brief, the measured ASL signal for a certain LD/PLD in a given voxel *r*_*i*_ is a sum of contributions from individual tissue types: $∆M\left(r_{i}\right)=\sum_{j=1}^{3}P_{j}\left(r_{i}\right)\cdot ∆m_{j}\left(r_{i}\right)$, where *P*_*j*_ is a row vector containing the tissue type fractions of this voxel (*j* = 1 is GM, *j* = 2 for WM, and *j* = 3 for CSF) as taken from the PVs, and ∆*m*_*j*_(*r*_*i*_) is a column vector of the signal intensities for each tissue type. This makes an underdetermined system with three unknowns (∆*m*_GM_, ∆*m*_WM_, ∆*m*_CSF_) and only one measurement. To solve this, we assume that ∆*m*_*j*_ is constant over a small kernel of *n* × *n* voxels (in plane) centered at *r*_*i*_ (represented as ${\bar{∆m_{j}\left(r_{i}\right)}}_{j}$) and the equation becomes $∆M\left(r_{k}\right)=\sum_{j=1}^{3}P_{\mathrm{kj}}\cdot {\bar{∆m_{j}\left(r_{i}\right)}}_{j}$, with $∆M\left(r_{k}\right)$ a column vector containing the *n* ^2^ signal values for all voxels within the kernel, and $P_{\mathrm{kj}}$ an *n* ^2^ × 3 matrix with the tissue probabilities for the three tissue types in each voxel in the kernel. For a kernel of 5 × 5 voxels in plane, this results in a system of 25 equations for three unknowns, with the solution

$$\bar{∆m}={\left(P^{T}\cdot P\right)}^{-1}\cdot P^{T}\cdot ∆M$$

which gives an estimate of the pure GM, WM, and CSF signal in every voxel.

#### 2.3.4 Estimating errors and differences in PVC results

To compare the results of PVC with and without the inclusion of CSF, we focused on the effect on GM CBF (as it borders with CSF and is the most relevant and reported ASL perfusion parameter). In the simulation dataset, the error on the GM signal was obtained by computing the relative difference (as a percentage) between the PV‐corrected GM signal and the ground truth. This was done for PVC with (CSF‐PVC) and without CSF (non‐CSF‐PVC), where the CSF signal is assumed to be zero. In the case of the in vivo data, no ground truth is known, so the relative difference between CSF‐PVC and non‐CSF‐PVC was calculated in lieu of the error. The PVC‐CBF was computed using both single PLD and multiple LD/PLD data: for single PLD, the data with LD/PLD = 2/2 s was employed with the equation found in Reference 17 used for quantification; for multiple LD/PLD time points, PVC was first applied to each LD/PLD dataset to extract pure GM ASL signal and subsequently this signal was fitted with a Buxton model to retrieve the CBF.

To estimate the size of the effect of CSF signal as a function of GM probability, regions of interest (ROIs) were created from the GM TPM for bins of 10% GM probability: i.e., the first ROI contains all voxels with GM_prob_ = 0–10%, the second GM_prob_ = 10–20%, etc. Since we are interested specifically in the interaction between GM and CSF, all voxels that contained more than 30% WM were eliminated from these ROIs.

We also studied the effects of PVC in vivo in the choroid plexus specifically. An ROI was created by delineating the posterior part of the lateral ventricles in three slices where the choroid plexus is located, starting from a CSF mask with a low PV threshold of 30%. This ROI therefore contains both the choroid plexus itself and a margin of CSF that immediately surrounds it.

Finally, to determine whether CSF signal estimation from PVC could act as a surrogate for long‐TE acquisitions to isolate pure CSF signal, PVC‐CSF signal was averaged over an ROI of the subarachnoid space for each LD/PLD pair, and similarly the long‐TE signal was averaged over the same ROI (summing the signal of the last three TE values, 1315–1837 ms). The time courses of these signals were plotted for comparison.

## 3 RESULTS

### 3.1 Results in the simulated dataset

**FIGURE 2:**
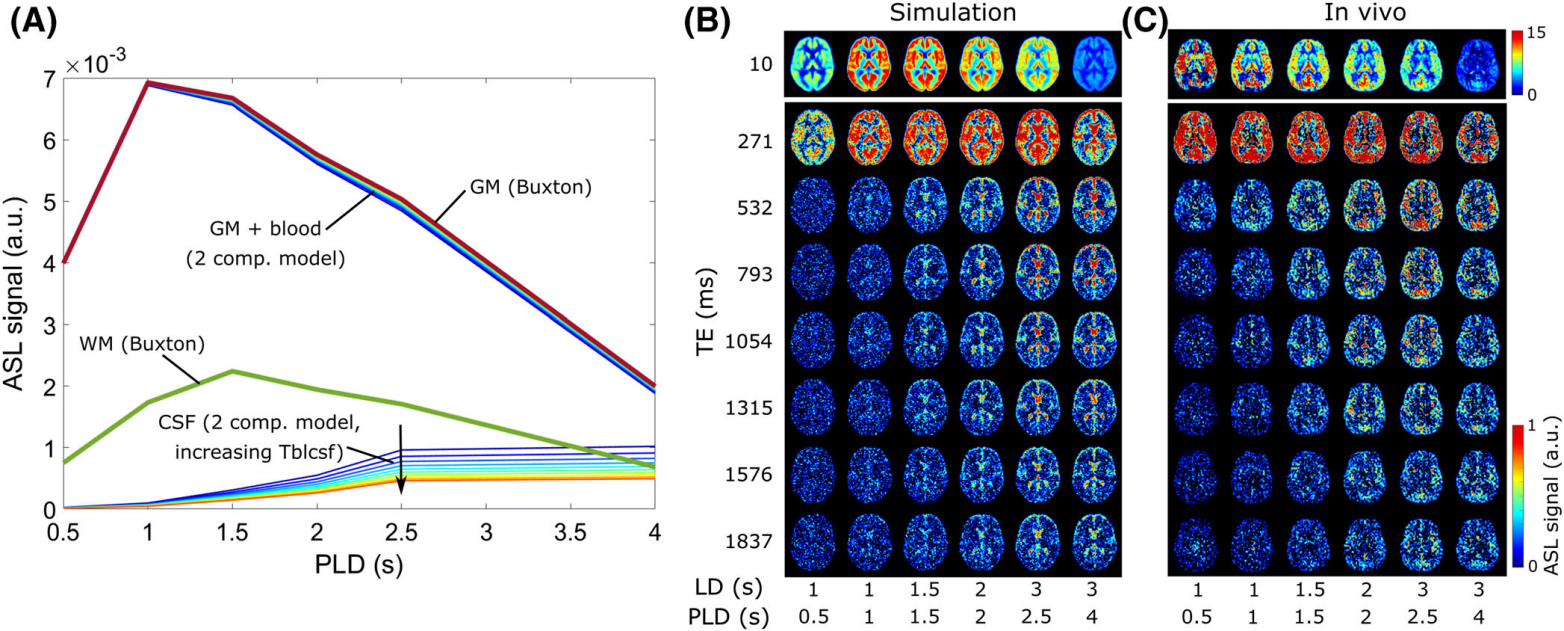
Results of the simulated data. A, Pure GM and WM (bold lines) and CSF signals. GM and WM values are obtained from Buxton modeling. Thin lines represent either the GM + blood or CSF compartment signal obtained from the two‐compartment dynamic model for 10 different values of *T*_bl→CSF_ (increasing from dark blue to red). All signals are modeled for TE = 0 ms. B, Resulting signal maps in a single slice. C, The corresponding in vivo data from a single subject.

The simulated data are shown in Figure 2. In Figure 2A, the time courses of the pure signal from each compartment (GM, WM, and CSF) are given. These values are used to create the signal maps shown in Figure 2B, which are contrasted with the in vivo signal in one subject in Figure 2C. We observe good visual agreement between these datasets.

**FIGURE 3:**
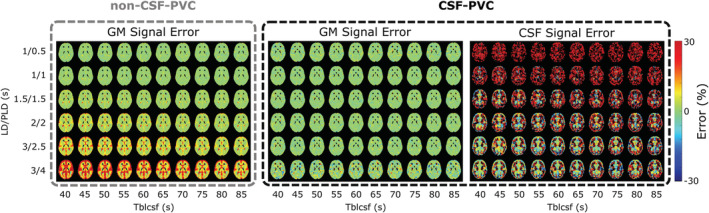
Maps of relative error from ground‐truth signal as obtained from the simulation study. GM signal errors without and with using PVC are shown in the left and middle panels, and error on the CSF signal (from CSF‐PVC) is shown on the right.

The signal maps from the simulated data were subsequently processed with both non‐CSF‐PVC and CSF‐PVC. The relative error on the resulting GM signal compared with ground truth is shown in Figure 3 (left and middle) as well as the error on the CSF signal (right) for a single slice intersecting the choroid plexus. A substantial error in non‐CSF‐PVC appears in some GM voxels surrounding the ventricles at the LD/PLD 1.5/1.5 s time point, and increases as CSF signal increases with longer LD/PLDs. The error is highest (reaching ~30%) in areas containing large amounts of CSF (ventricles, subarachnoid space) and when *T*_bl→CSF_ is shortest. When CSF is included in the PVC (middle), the error on the GM signal is largely resolved. The CSF signal error (right) is large at early time points, and as we move toward longer time points the signal is first strongly affected by noise levels, then becomes more stable, and the error is finally shown to be small and centered around zero.

**FIGURE 4:**
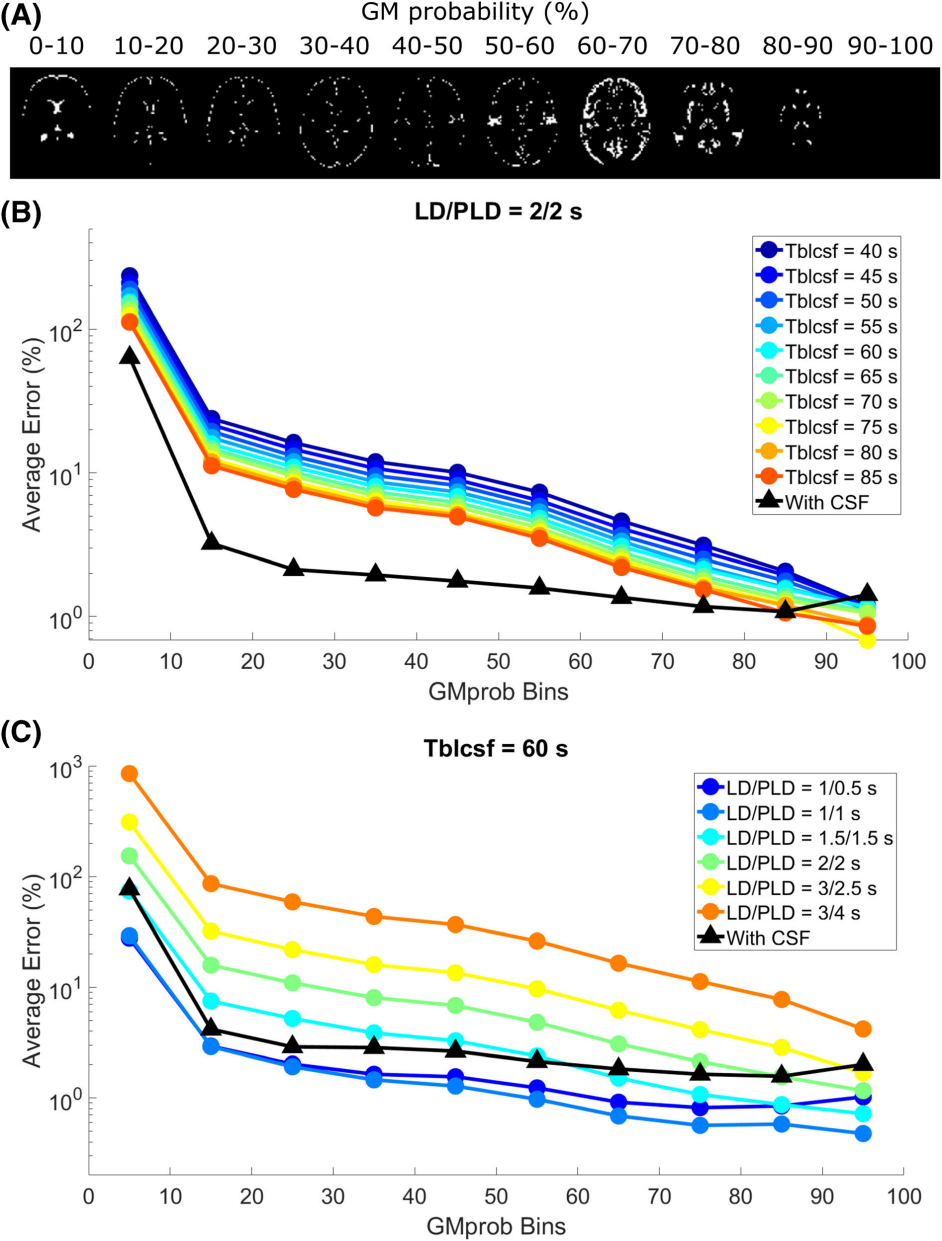
Average error on the GM signal in simulations. A, GM_prob_ ROIs for a single slice. B, C, Average error for these ROIs for a single time point and all *T*_bl→CSF_ values (B) and for all time points and a single *T*_bl→CSF_ value (C). The errors for non‐CSF‐PVC (colors, circles) are contrasted to the error when using CSF‐PVC (black, triangles).

To average and compare this error, voxels were binned according to their GM probabilities. These ROIs are shown in Figure 4A for a single slice. In Figure 4B, the average error as a function of GM_prob_ ROI is shown for a single LD/PLD of 2/2 s (closest to the recommended pCASL settings used in the clinic for perfusion imaging) and all *T*_bl→CSF_ values. Without CSF correction (colors, circles), the error consistently exceeds the error when CSF is included (black, triangles), but the difference decreases with increasing GM_prob_, and for voxels with 90% GM or more the results are similar, with the error approaching zero. CSF‐PVC yielded results that were very similar for all *T*_bl→CSF_ values and LDs/PLDs; therefore, we show only one curve instead of several overlapping curves. In Figure 4C, the same results are given for a single *T*_bl→CSF_ value of 60 s (the average value found in vivo) and all time points. For the two earliest time points, there is no improvement in using CSF‐PVC, as the low CSF signal only introduces additional error due to the presence of noise in the absence of signal. For LD/PLD = 1.5/1.5 s, the effect of using CSF‐PVC is mixed, as only voxels with less than 50% GM see an improvement in GM signal estimation. For LD/PLD = 2/2 s and higher, the reduction in error from using CSF‐PVC is substantial and reaches up to 100%.

**FIGURE 5:**
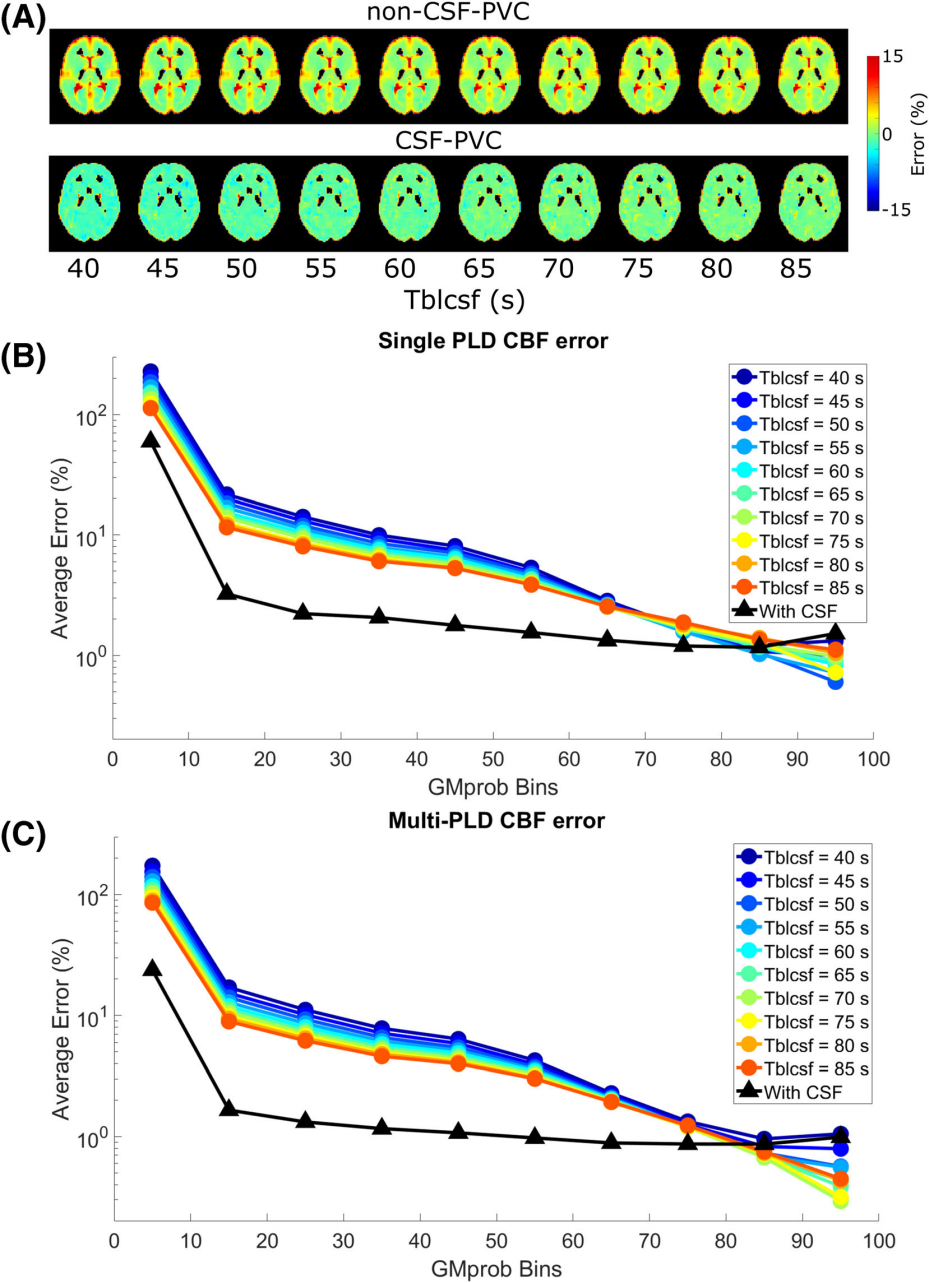
Relative error on CBF quantification with two methods in the simulated dataset. A, B, CBF quantified with a single LD/PLD of 2/2 s. Error is shown as maps (A) and averages over ROIs (B, C, ROIs from Figure 4A). C, CBF quantified by fitting signal from all time points to a Buxton model.

Next, CBF was quantified from the PV‐corrected GM signal, first by using the single PLD equation given in the ASL white paper^17^ for LD/PLD = 2/2 s (closest to the recommended pCASL implementation), and second by fitting the six time points (i.e., LD/PLD combinations) to a Buxton model. In Figure 5A, error maps using the single‐PLD CBF approach are shown for non‐CSF‐PVC and CSF‐PVC. In certain areas of the brain, non‐CSF‐PVC leads to overestimation of the CBF by as much as 15%. When CSF is included in the PVC algorithm, the error is much more uniform, but there is a slight underestimation of CBF across all voxels, as is typical for single‐PLD CBF quantification. When averaging over GM probability bins (Figure 5B), these effects compete, leading to a smaller error in single‐PLD CBF quantification with non‐CSF‐PVC than with multi‐PLD CBF quantification with non‐CSF‐PVC (Figure 5C). Even though, in both single‐PLD and multi‐PLD CBF, the error is consistently lower when using CSF‐PVC than non‐CSF‐PVC, the improvement for single‐PLD quantification is more limited. The error is lowest when using multi‐PLD CBF quantification with CSF‐PVC.

### 3.2 In vivo results

3.2

**FIGURE 6:**
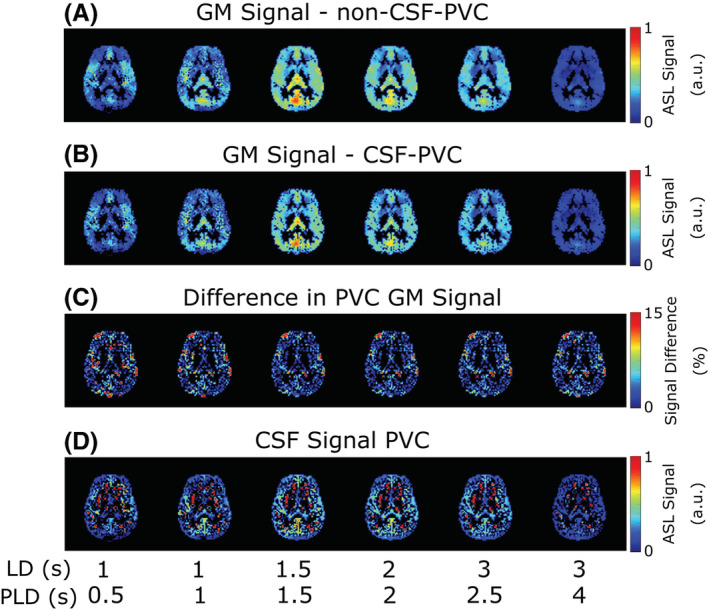
Maps of the results of PVC in vivo. A, GM signal with non‐CSF‐PVC; B, GM signal in CSF‐PVC; C, relative difference between them, for all LD/PLD pairs in a single slice. D, CSF signal obtained from PVC.

Figure 6 shows the results of the same procedure applied to in vivo data. In Figure 6, PVC GM signal is contrasted for non‐CSF‐PVC (A) and CSF‐PVC (B). Although these values are similar, differences appear in areas that coincide with the presence of CSF, such as the ventricles and subarachnoid space, where CSF‐PVC GM signal is noticeably lower. The third row (C) shows the relative difference (absolute values) between the two methods. A small difference appears and is well distributed around the cortex, with small regions exhibiting peaks of 15% difference or more in some regions of the subarachnoid space and around the choroid plexus in the ventricles. Finally, PVC ASL signal in the CSF is shown on the bottom row (D), with areas of higher CSF signal corresponding to areas of larger differences between CSF‐PVC GM signal and non‐CSF‐PVC GM signal, as expected. Areas containing little to no CSF (for example, close to the WM) display large, erroneous values for CSF signal, which can easily be explained by unstable and noise amplifying behavior of the PVC algorithm.

**FIGURE 7:**
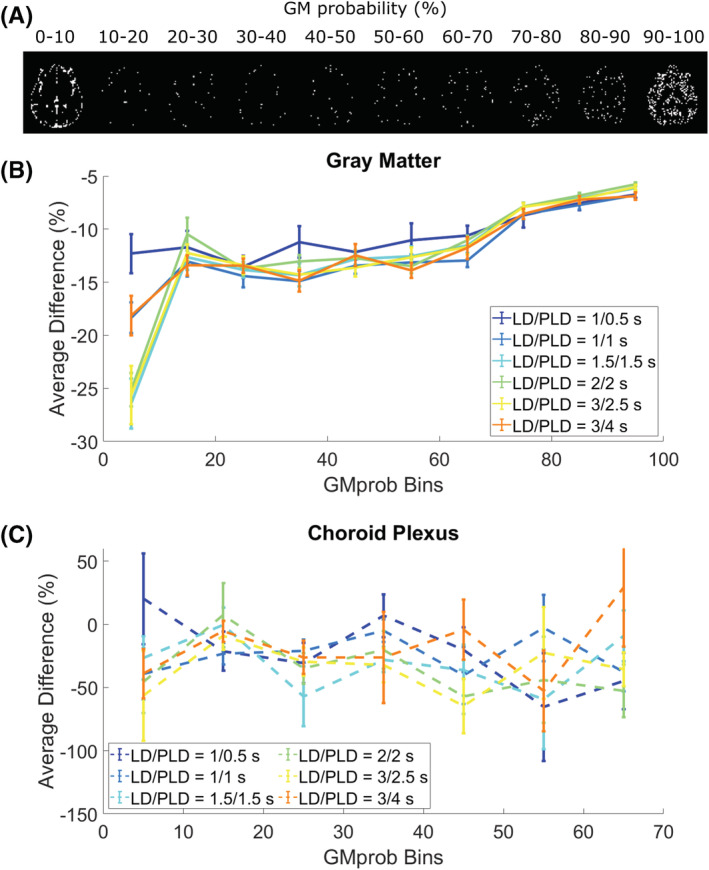
Results of PVC in vivo for different GM_prob_ (or PV) bins. A, The ROIs based on GM_prob_ for a single subject. B, Average difference between CSF‐PVC and non‐CSF‐PVC for ROIs based on GM_prob_ (group average ± standard error of the mean) for all time points. C, The same results for the choroid plexus ROI in isolation.

**FIGURE 8:**
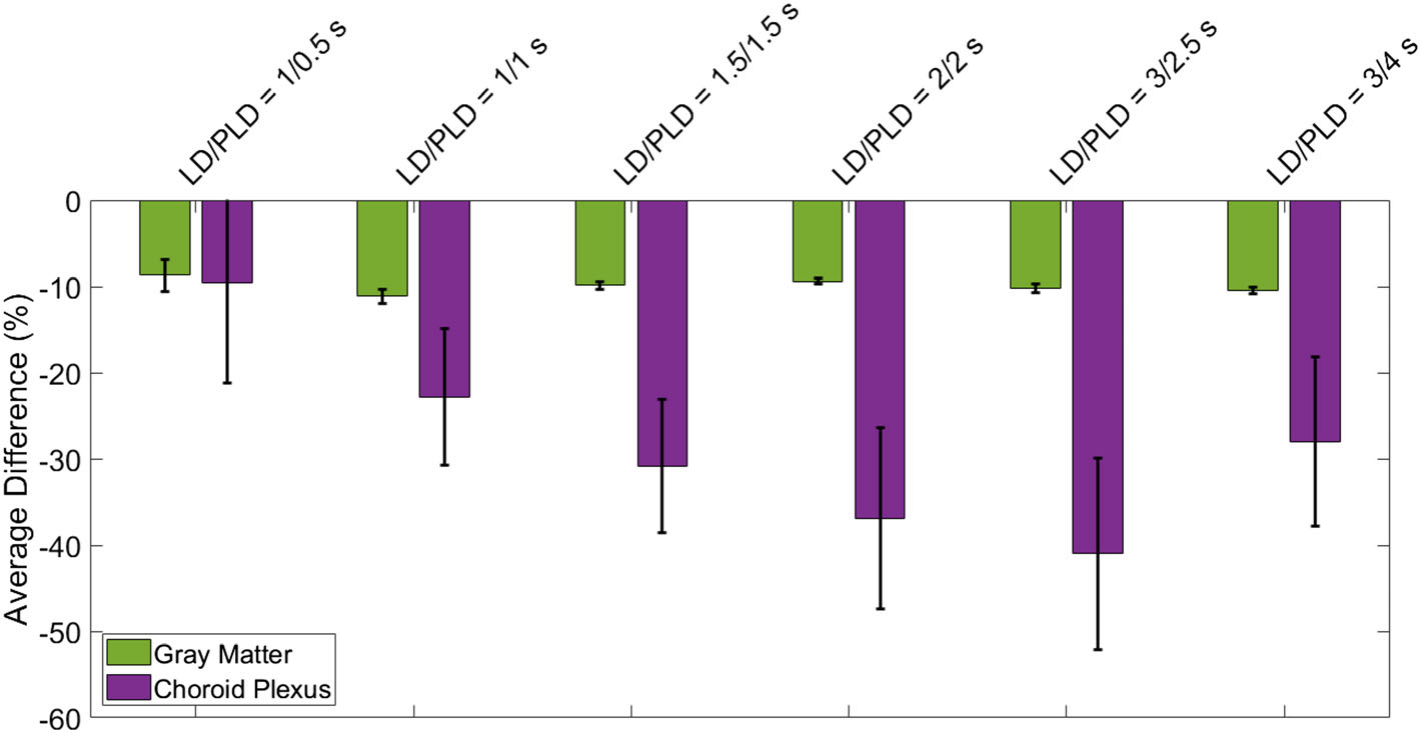
Differences between CSF‐PVC and non‐CSF‐PVC, averaged over all voxels of a GM ROI in a single slice high in the brain, and the choroid plexus ROI. The error bars represent the standard error of the mean over all subjects.

In Figure 7B, the average difference between the GM signal obtained with CSF‐PVC and non‐CSF‐PVC is plotted for ROIs corresponding to bins of GM_prob_ (shown in Figure 7A for a single subject), extracted in the same way as for the simulation data. These curves are averaged over all subjects (with error bars representing inter‐subject standard error of the mean). We see differences in excess of 10%, for voxels containing up to 70% GM, which is of the same order of magnitude as the error calculated in the simulated dataset. This suggests that correcting for the presence of CSF signal in PVC improves GM signal estimates by roughly 10%, fairly independently of LD and PLD. In the choroid plexus (Figure 7C), this difference is more uniform across GM_prob_ bins, and it is larger than in the rest of the GM, on average exceeding 30%. The choroid plexus ROI contains far fewer voxels than the overall GM; therefore, the variability across subjects is larger (leading to wider error bars). For direct comparison of the effect of CSF‐PVC in the choroid plexus versus in the GM, Figure 8 shows a bar graph of the average difference in the choroid plexus and GM ROIs. Whereas the average difference in the GM was around 10% and largely independent of LD and PLD, in the choroid plexus this difference is significantly higher, and varies with LD/PLD in a pattern resembling the time course of the CSF signal, peaking at a difference of more than 40% for an LD/PLD of 3/2.5 s.

**FIGURE 9:**
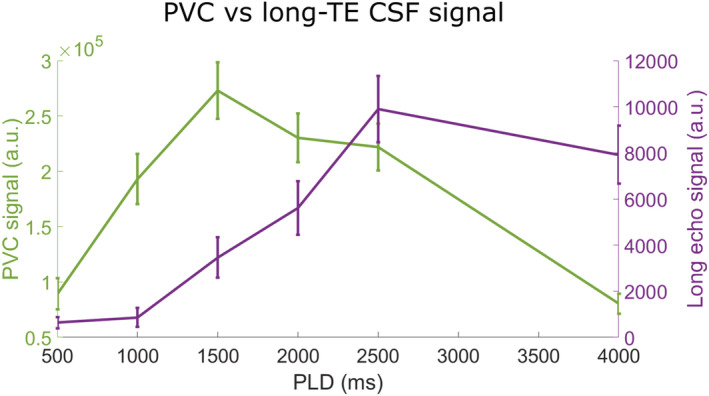
Comparison of the pure CSF signal through time obtained with long‐*T*_E_ acquisitions with CSF signal calculated with PVC.

In Figure 9, we compare the time course of the average PVC CSF signal in the subarachnoid space (green, left‐hand axis) with that of the average long‐TE signal in the same ROI (purple, right‐hand axis). If PVC‐CSF could be considered as an alternative approach to estimate the ASL signal in the CSF, we would expect similar shapes for these two curves. However, it is evident that they are not. Indeed, the PVC CSF signal peaks much earlier and exhibits a fast decay afterwards, whereas the long‐TE signal (which more accurately represents pure CSF) follows a slower upslope and later peak.

## 4 DISCUSSION

In this paper, we have investigated the effect of CSF signal on ASL PVC, used to extract pure perfusion parameters for given tissue types, and compared the real CSF signal time course from long‐TE acquisitions in vivo with the CSF signal extracted using PVC. Our main conclusions are that ASL signal in the CSF may lead to errors in GM‐CBF estimates of approximately 10% when CSF signal is neglected in PVC, and that CSF‐PVC cannot be used as an alternative to long‐TE ASL experiments for measuring water transport across the BCSFB from traditional ASL scans. The origin of these two seemingly conflicting conclusions will be the main topic of this discussion.

First, using simulated and in vivo data, we show that errors on pure perfusion parameters are reduced when CSF is included in the PVC algorithm. In simulated data, the error on the pure GM signal (Figures 3 and 4) is largest in conditions where more CSF signal is present, including areas of the brain with large CSF pools, longer LD/PLDs, and shorter *T*_bl→CSF_ exchange times. These results are to be expected, as when CSF is excluded from PVC the signal originating from this compartment is labeled as either GM or WM signal, and because of the close proximity of GM and CSF in the brain this will largely fall into the GM compartment. This effect will be most severe when more labeled spins are present in the CSF—i.e., for larger CSF PV, longer LD/PLD, and shorter *T*_bl→CSF_ (faster exchange of water from blood to CSF). This leads to systematic overestimation of pure GM signal in most GM‐containing voxels, which is largely corrected by the inclusion of CSF in PVC. For the clinical ASL parameters of LD/PLD = 2/2 s, the error is consistently lowered in CSF‐PVC for voxels with 90% GM or less. For earlier time points, this improvement is limited, especially due to the fact that the CSF‐ASL signal becomes small compared with the noise, and for longer time points it is increased. This is particularly visible in Figure 3, where large errors caused by noise are present in the PVC‐CSF signal for short LDs/PLDs and long *T*_bl→CSF_.

In vivo, the difference between non‐CSF‐PVC and CSF‐PVC GM signal exceeds 10% in most voxels (Figure 7B), which is of the same order of magnitude as the improvement seen in simulations. Additionally, this same difference estimated in an ROI surrounding the choroid plexus (Figure 7C) is much larger, averaging approximately 30%. When looking at the whole ROI (Figure 8), the average difference in the GM is about 10% independent of LD and PLD, and in the choroid plexus it varies with LD/PLD between 10% (shorter LD/PLD) and 40% (longer LD/PLD). This leads us to conclude that including CSF in PVC to extract pure GM signal, which is a fairly straightforward extension of the traditional two‐component PVC technique, leads to more accurate estimates of GM signal, particularly in the choroid plexus, where the difference between these techniques is heightened. When studying choroid plexus perfusion difference in ageing, CSF‐PVC will especially be important for proper interpretation.^7^ On the other hand, when quantifying CBF, other effects influence the PVC parameters. As seen in the simulation, multi‐PLD CBF quantification with the Buxton model (Figure 5C) also benefits from reduced error when including CSF signal; however, for single‐PLD quantification (Figure 5A and 5B), the benefits are slightly more limited. This is because our single‐PLD quantification underestimates slightly the true CBF, while non‐CSF‐PVC leads to overestimation. These effects compete to result in improvements in quantification that are smaller for single‐PLD quantification than for multi‐PLD; however, CBF estimates are always improved by CSF‐PVC. We note that the increased accuracy of CBF quantification with single‐PLD non‐CSF‐PVC compared with multi‐PLD (i.e., comparing colored lines in figure 5B versus 5C) is purely coincidental and not the result of a better quantification method.

Our second aim was to determine whether CSF signal can be accurately estimated from PVC, without the need for long‐TE acquisitions to isolate the CSF signal by taking advantage of the much longer *T*_2_ of CSF. This would also have the added advantage that CSF signal and thus water transport measurements across the BCSFB could be extracted retroactively from existing ASL data, potentially providing more insight into brain clearance mechanisms. To determine this, we compared the time course of the CSF signal as measured in long‐TE ASL (Figure 7, purple curve) with the PVC CSF signal (green curve). The difference between these curves is immediately apparent. CSF signal from PVC applied to the first echo time data displays an earlier peak followed by a quick decay of the signal. When this same comparison is done on the simulated data (not shown), the two curves exhibit much more similar behaviors than in vivo. This is because a key difference between our simulation and in vivo datasets is that macrovascular signal was not included in the simulation study. In vivo, ASL signal remains present in the arteries especially for the early time points, and the position of many of these arteries coincides with areas containing CSF.^18^ Segmentation of the arterial tree is not performed in this protocol, since anatomical differences between subjects are large and no standardized arterial probability map was available in SPM12. Since the PVC algorithm estimates the GM, WM, and CSF PVs in each voxel based purely on their relative volume fraction without including an arterial component, intravascular signal can bleed into the other components and thereby overwhelms the actual CSF‐ASL signal without additional information. We include a step in image preprocessing to remove this macrovascular component, aBV, by fitting the data to a perfusion model which includes this component, and then subtracting it from the original data. This method is effective at removing a large fraction of the macrovascular signal, but not all of it. We tried a number of techniques to more accurately estimate and remove this signal (for example, by fitting the perfusion and macrovascular blood signal separately or together), as well as more aggressive techniques such as masking out all signal from the arteries (thresholding the first LD/PLD time point to create this arterial mask). In all of these cases, residual arterial signal could still be identified. We therefore conclude that, unfortunately, CSF signal cannot be accurately quantified by employing PVC only. Other acquisition methods may be more effective in estimating and/or removing macrovascular signal, such as the use of crusher gradients in the imaging sequence, which physically removes intravascular blood signal before readout^9^; alternatively, acquiring a higher number of short‐LD/PLD images to better sample the arrival of blood in the vasculature would lead to more accurate estimates of aBV.^19^, ^20^

The two principal findings of this study appear to show some disagreement. We show both that including CSF signal in PVC improves ASL quantification for GM perfusion parameters, and that the PVC CSF signal in vivo does not accurately represent pure CSF signal. However, in reality, these results are not contradictory and do point to the usefulness of implementing CSF‐PVC. As stated above, we attribute the discordance to contamination of the CSF signal with macrovascular blood signal. The added freedom provided by the three‐component PVC algorithm results in two sources of signal (macrovascular and CSF) becoming entangled as a result. Nevertheless, both of these contributions should not be attributed to GM when performing CBF quantification, and PVC should benefit from the inclusion of these components. Therefore, the CSF‐PVC approach remains optimal, even though it does not limit its correction to CSF signal. This statement is also supported by simulation studies showing that the CSF‐PVC approach results in improved GM‐CBF measurements in the absence of macrovascular signal, and that in this case the CSF signal is also successfully isolated.

Moreover, our results show that the choroid plexus is particularly sensitive to PVC and that the inclusion of CSF signal in this correction will be crucial when measuring perfusion in this small brain region. As there is growing interest in brain clearance mechanisms, and specifically in CBF measurements in the choroid plexus, we believe that the CSF‐PVC method adds value and removes some bias from these measurements. This is true especially when looking at the glymphatic system in the aging brain. While it has been shown that CSF and perivascular space volume increase with age,^21^, ^22^, ^23^, ^24^, ^25^, ^26^, ^27^ and average GM CBF decreases,^28^, ^29^, ^30^, ^31^, ^32^ studies on the relationship between CSF flow, blood–CSF water exchange and age are more rare, often only done in animals, and sometimes lead to contradicting conclusions.^33^, ^34^, ^35^, ^36^, ^37^, ^38^ CSF‐PVC may be a critical tool in disentangling the contributions from numerous effects that alter brain clearance mechanisms in the aging brain.

There are a number of limitations to this study that warrant discussion. First, the correspondence between the in vivo and simulated datasets was not perfect. For one, the simulation data are obtained simply by multiplying the pure GM, WM, and CSF signals by their corresponding TPMs, meaning that CSF signal appears in all CSF areas, and not only the ones where actual exchange is present. For example, there is signal in the frontal horns of the ventricles in the simulation, whereas this is not the case in vivo, as there is no choroid plexus in that region, and no other source of blood‐to‐CSF water exchange. In vivo, all the CSF signal, whether detected in the ventricles near the choroid plexus or near arteries within the subarachnoid space, is the result of exchange in the immediate vicinity of the signal, i.e., within the voxel, because of the short time frame of measurement (a few seconds of labeling and PLD), which does not allow for large‐scale movement of the ASL signal to spread through the CSF compartment by diffusion or flow. As we have already discussed, the simulation also did not include macrovascular blood signal, while the in vivo data did. This explains the surprising result that the orders of magnitude (~10%) of both the effect of CSF signal in PVC in simulations and the difference between CSF‐PVC and non‐CSF‐PVC in vivo are so similar, when we show that these do not represent the same signal (i.e., purely CSF signal in simulations versus CSF + macrovascular signal contamination in vivo). In reality, our simulation overestimates the effect of CSF signal, because simulated maps assume that CSF‐ASL signal appears everywhere that CSF is present, while in vivo CSF signal only appears where exchange of water from blood to CSF occurs. Therefore, the overestimation of CSF signal in simulations appears to be of the same order of magnitude as the overestimation due to macrovascular contamination in vivo. Additionally, our simulation is a simplified model where only GM, WM, and CSF signals are present, so naturally the PVC algorithm, which also assumes only three compartments, retrieves ground‐truth values with greater accuracy than it would in vivo, where other sources of signal and artefacts may be present. Finally, we noticed that the ROIs based on GM_prob_ differed between the simulation (Figure 4A) and in vivo (Figure 6B). In the latter case, the ROIs were much more skewed towards the extremes, either containing very little GM (0–10%) or a lot (90–100%), therefore the error/difference curves may not be entirely equivalent.

In the scope of this study, we investigated the effects of CSF only on linear regression PVC algorithms; however, other more complex versions of PVC have also been developed—for example, based on information from multi‐PLD acquisitions and statistical models^2^ or Look–Locker sequences.^39^ It remains to be investigated whether these techniques would also benefit from including CSF signal, and if those models lead to better separation of the CSF and macrovascular blood signal.

## 5 CONCLUSION

Including CSF in PVC leads to an improvement in pure GM signal estimation of approximately 10%, with larger differences, reaching up to 40%, observed in the choroid plexus. This improvement carries over to multi‐PLD CBF quantification; however, for single‐PLD CBF competing effects lead to more limited gains from CSF‐PVC. We also show that PVC CSF signal is not a substitute for long‐TE CSF signal, as it suffers from macrovascular blood signal contamination. We would therefore recommend the use of CSF‐PVC, especially when measuring perfusion in the choroid plexus.
